# The role and diagnostic accuracy of serology for COVID-19

**DOI:** 10.1186/s12879-022-07361-y

**Published:** 2022-04-19

**Authors:** Debasree Kundu, Priyanka Gautam, Divya Dayanand, Karthik Gunasekaran, Abi Manesh, Merylin Sebastian, Kundavaram P. P. Abhilash, Anand Zachariah, Tina George, Sowmya Sathyendra, Samuel G. Hansdak, O. C. Abraham, Ramya Iyadurai, Balamugesh Thangakunam, Richa Gupta, Rajiv Karthik, Mahesh Moorthy, George M. Varghese

**Affiliations:** 1 Department of Infectious Diseases, Christian Medical College, Vellore, 632004 Tamil Nadu India; 2 Department of Medicine, Christian Medical College, Vellore, Tamil Nadu India; 3 Department of Emergency Medicine, Christian Medical College, Vellore, Tamil Nadu India; 4 Department of Pulmonary Medicine, Christian Medical College, Vellore, Tamil Nadu India; 5 Department of Respiratory Medicine, Christian Medical College, Vellore, Tamil Nadu India; 6 Department of Clinical Virology, Christian Medical College, Vellore, Tamil Nadu India

**Keywords:** COVID-19, SARS-CoV-2, Antibody test, IgM, IgG, Serology

## Abstract

**Background:**

The role and performance of various serological tests for the diagnosis of COVID-19 are unclear. This study aimed to evaluate the performance of seven commercially available serological assays for SARS-CoV-2 antibodies by testing COVID-19 cases and controls.

**Methods:**

Adult patients with fever for > 5 days, admitted to a tertiary-care teaching hospital in South India, were enrolled prospectively between June and December 2020. SARS-CoV-2 RT-PCR confirmed patients were classified as cases, and patients with febrile illness with laboratory-confirmed alternative diagnosis and healthy participants were controls. All participants were tested with SCoV-2 *Detect*™ IgM ELISA kit and SCoV-2 *Detect*™ IgG ELISA kit (InBios International, Seattle, USA) (Inbios), SARS-CoV-2 Total and SARS-CoV-2 IgG (Siemens Healthcare Diagnostics Inc., Tarrytown, USA) (Siemens), Roche Elecsys® Anti-SARS-CoV-2 (Roche Diagnostics, Rotkreuz, Switzerland) (Roche), Abbott SARS-CoV-2 IgG (Abbott Diagnostics, IL, USA) (Abbott), and Liaison® SARS-CoV-2 S1/S2 IgG (DiaSorinS.p.A., Saluggia, Italy) (Liaison). The sensitivities, specificities, positive predictive values (PPV), negative predictive values (NPV), and accuracies were compared.

**Results:**

There were 303 participants: 153 cases and 150 controls. ELISA detecting anti-S protein antibody was more sensitive (88.9% for IgG and 86.3% for IgM) than the CLIAs (82.4% for total antibodies and 76.5–85.6% for IgG). Among CLIAs, Roche IgG was most sensitive (85.6%) followed by Abbott (83%) and Liaison (83%). Abbot had the best PPV (88.8%) and was more specific (89.3%) than Liaison (82%) and Roche (82%). Siemens IgG was less sensitive (76.5%) than Siemens Total (82.4%). The specificity of all the serological assays was modest (75–90%). Antibody test positivity increased with the duration of illness reaching 90% after 10 days of illness. When cases were compared against pre-pandemic controls, the IgG gave excellent specificity (98–100%). For seroprevalence studies, InBios IgG had the best accuracy (90.8%) with 88.9% sensitivity and 97.6% specificity.

**Conclusion:**

The serological assays are important adjuncts for the diagnosis of COVID-19 in patients with persistent symptoms, especially in the second week of illness. The value of serological diagnostic tests is limited in the first week of illness and they provide additional value in seroprevalence studies. The diagnostic accuracy of the ELISA and CLIA platforms were comparable.

## Background

The novel coronavirus disease (COVID-19) pandemic poses huge challenges to the already stretched health care systems in India and several other countries. In the wake of this highly contagious viral disease, over 398 million people were confirmed to have novel severe acute respiratory syndrome coronavirus (SARS-CoV-2) infection resulting in over 5.5 million deaths worldwide as of Feb 2022 [1].

The large majority of patients (about 80%) presents with mild disease and recover within a week [2]. They do not require diagnostic evaluation. However, about 15% of patients develop persisting symptoms progressing to moderate or severe disease with complications including lower respiratory involvement a week after the onset of symptoms [3, 4]. Currently, the detection of the SARS-CoV-2 viral RNA by real-time reverse transcriptase-polymerase chain reaction (RT-PCR) remains the gold standard test for the diagnosis of COVID-19 [5]. However, it is expensive and demands sophisticated laboratory facilities and expertise. Further, the sensitivity of its results depends on the type and adequacy of sample, sampling technique, time of sample collection in relation to symptom onset, and viral load [6, 7].

Serological tests that detect IgM, IgG, IgA or total antibodies against SARS-CoV-2 are cheaper and easier to perform [8]. It has also been established that seroconversion occurs in vast majority of individuals in 10–14 days after the onset of symptoms [9]. Hence, among patients with persisting symptoms, severe disease, or complications, antibody detection may aid in the cost-effective diagnosis. Serological tests can also aid in population-based seroprevalence studies.

Several serological assays are currently commercially available that differ in format (lateral flow immunoassays (LFA), enzyme-linked immunosorbent assays (ELISA) and chemiluminescent immunoassays (CLIA) and SARS-CoV-2 antigen employed in assay design (recombinant nucleocapsid protein (NP), subunit 1 of the spike glycoprotein (S1), the Spike glycoprotein receptor-binding domain (RBD), etc. However, the role and accuracy of serologic assays are not adequately evaluated which calls for evaluation of each assay to validate their clinical utility.

This study evaluated the performance of serological tests of different formats that detect IgM, IgG, and total antibodies against SARS-CoV-2 antigens by testing well-characterized COVID-19 cases and control.

## Materials and methods

Patients older than 18 years of age with symptoms of fever or respiratory illness of more than 5 days duration who were admitted to a tertiary care teaching hospital in South India were enrolled prospectively between June 2020 and December 2020 after obtaining informed consent. Pre-pandemic controls were collected prior to January 2020. This study was approved by Institutional Review Board and Ethics Committee of Christian Medical College, Vellore (No.13166/22.07.2020).

A detailed history and physical examination were carried out and documented using a predesigned proforma. As per routine practice, patients were investigated and managed by the attending physician for common febrile illnesses such as dengue, scrub typhus, malaria, typhoid and COVID-19.

Patients confirmed to have COVID-19 with a positive real-time RT-PCR (RT-PCR) for SARS-CoV-2 on a nasopharyngeal sample taken at admission were considered as cases. Patients negative for SARS-CoV-2 by RT-PCR with acute undifferentiated febrile illness and laboratory-confirmed alternate etiologies including culture-positive enteric fever smear or PCR-positive malaria, dengue PCR or NS1 antigen positive, and scrub typhus 47 kDa htrA real-time PCR positive were considered as controls. Healthy controls consisted of asymptomatic subjects from the same geographic region recruited during the same period. Healthy volunteers and controls with similarly confirmed etiologies who presented before January, 2020 were also included and characterized as pre-pandemic controls.

A 5 ml blood sample was collected from all patients, in sterile EDTA tubes, centrifuged to separate plasma and buffy coat, and stored at − 80 °C until further testing. Serological assays using ELISA and CLIA to detect IgM, IgG and total antibodies were done on all samples to assess the performance of each test. The following serological assays were included in this study.

### Enzyme linked immunosorbent assay (ELISA)

SCoV-2 *Detect*™ IgM ELISA kit and SCoV-2 *Detect*™ IgG ELISA kit (InBios International, Seattle, USA) is an in vitro, qualitative, indirect ELISA test used to detect the presence of IgM and IgG antibodies respectively against SARS-CoV-2 S proteins. The incurred sample reanalysis values above 1.1 are considered as positive.

### Chemiluminiscence immunoassay (CLIA)

SARS-CoV-2 Total (COV2T) and SARS-CoV-2 IgG (COV2G) are chemiluminescent immunoassays to detect the presence of total antibodies (IgM, IgG) and IgG, respectively in human serum and plasma and were performed using the ADVIA Centaur® XP System (Siemens Healthcare Diagnostics Inc., USA). The assay uses the Spike-Receptor binding domain (S1-RBD) as antigen. A cut-off-index (COI) of 1 or more is considered positive.

Roche Elecsys® Anti-SARS-CoV-2 (Roche Diagnostics, Switzerland) is an electrochemilumiscent immunoassay for the detection of antibodies to SARS-CoV-2 nucleocapsid (N) protein and performed on the Cobas® e401 analyser. A COI of 1 or more is considered reactive.

The Abbott SARS-CoV-2 IgG (Abbott Diagnostics, IL, USA) is a CLIA for the qualitative detection of antibodies to the Nucleocapsid (N) protein of SARS-CoV-2 and performed on Abbott ARCHITECT™ *i*2000SR system. This assay is a qualitative detection of IgG antibodies against the SARS-CoV-2 in serum or plasma. A value of Sample/Cut-off (S/Co) < 1, 1.0–1.4 and ≥ 1.4 were considered negative, gray zone reactive; and positive respectively.

The LIAISON® SARS-CoV-2 S1/S2 IgG (DiaSorin, Italy) is a CLIA for detection of antibodies against the spike protein (S1/S2 subunits) and performed on the LIAISON® XL Analyzer. Sample results < 12, 12–15 and ≥ 15 arbitrary units were considered negative, equivocal and positive, respectively.

All assays were performed as per manufacturers’ instruction and result interpretation as per kit insert.

### Statistical analysis

The diagnostic serological tests for COVID-19 (ELISA and CLIA) were assessed for sensitivities, specificities, positive predictive values (PPV), negative predictive values (NPV), likelihood ratios, and accuracies [with 95% confidence intervals (CI)]. All Statistical analysis was performed using SPSS (IBM® SPSS Statistics version 14.1).

## Results

**Fig. 1:**
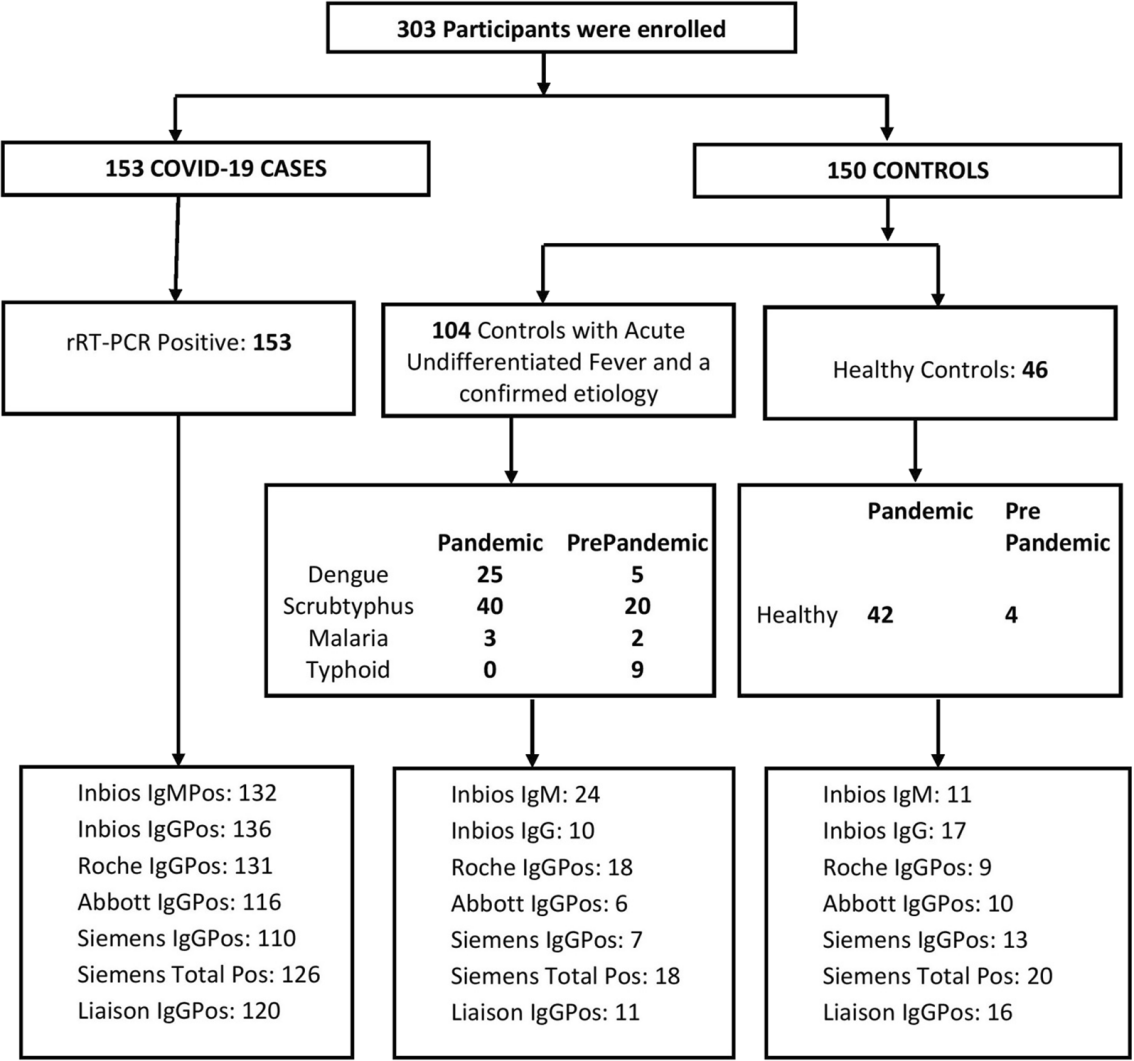
Schematic representation of the study showing positive results of each of the serological tests

A total of 303 participants, 153 cases and 150 controls, were included in this study. The details are presented in Fig. 1. All 153 cases were tested positive with the reference test, RT-PCR for SARS-CoV-2. Among the controls, 104 participants were diagnosed to have an acute febrile illness with a confirmed etiology and 46 were healthy controls.

Of the controls with acute febrile illness, 36 were diagnosed before January 2020 and were included as pre-pandemic controls and 68 were diagnosed during the study period of the pandemic. Of the healthy controls, four were volunteers included before January 2020 and counted as pre-pandemic controls to give a total of 40 pre-pandemic controls.

Participants with acute febrile illness were diagnosed with dengue (30), malaria (5), typhoid (9), or scrub typhus (60). Among them, 59 participants who were diagnosed during the pandemic period had tested negative for SARS-CoV-2 RT-PCR in addition to having confirmed alternate etiologies. The remaining participants with acute febrile illness, including nine who were diagnosed during the pandemic period, only had confirmation of alternate etiology without SARS-CoV-2 RT-PCR being performed. The SARS-CoV-2 RT-PCR was not performed on healthy controls and pre-pandemic controls.

The mean age of the participants was 48.2 years, and 55.4% of them were men. The patient characteristics are elaborated in Table 1. Among the cases, 68 (44%) were mild, 61 (33%) moderate, and 24 (16%) severe grades of disease.

**Table 1:** Patient characteristic of the participants in the serological assays

Patient characteristics	Cases (N = 153)	Controls (N = 150)
Age, Years, Mean ± SD	54.27 ± 13.92	42.32 ± 16.71
Sex, Male, n (%)	96 (62.7)	72 (48)
Duration of illness before admission, days, Mean ± SD	13.39 ± 3.84	8.49 ± 3.62
Fever present at admission, n (%)	135 (88.2)	104 (100)
WBC Count, cells/mm^3^, Mean ± SD	8336 ± 6272	10,186 ± 9979
Platelet Count, cells/mm^3^, Mean ± SD	267,927 ± 107,859	86,104 ± 72,643
Total Bilirubin, mg/dl, Mean ± SD	0.58 ± 0.34	2.12 ± 2.19
Direct Bilirubin, mg/dl, Mean ± SD	0.28 ± 0.23	1.71 ± 1.96
Total Protein, g/dl, Mean ± SD	7.48 ± 5.89	6.25 ± 1.04
Albumin, g/dl, Mean ± SD	3.93 ± 2.63	2.97 ± 0.78
AST, IU/ l, Mean ± SD	34.68 ± 19.88	181.38 ± 261.87
ALT, IU/ l, Mean ± SD	35.57 ± 25.38	103.38 ± 92.47
Alkaline Phosphatase, IU/ l, Mean ± SD	85.54 ± 45.68	169.68 ± 108.89
Serum Creatinine, mg/dl, Mean ± SD	1.11 ± 1.64	1.66 ± 1.75

*SD* standard deviation, *N* sample size, *AST* aspartate transaminase, *ALT* alanine transaminase

The values shown are based on the available data. Laboratory values for white-cell count (WBC), platelet count and serum creatinine were available for 150 cases and 103 controls; the values for total bilirubin, direct bilirubin, total protein and albumin values and alkaline phosphatase values were available for 147 cases and 101 controls; aspartate aminotransferase (AST) and alanine aminotransferase (ALT) values were available for 152 cases

The mean duration of illness before the sample collection was 13.4 days for cases and 8.49 days for controls. A higher level of total leucocyte count (TC) was seen among the controls (TC: 10.186 ± 9.979 × 10^9^/L) as compared to the cases (TC: 8.336 ± 6.272 × 10^9^/L). Similarly, lower platelet counts were noted among the controls than cases (86.104 ± 72.643 × 10^9^/L verses 267.927 ± 107.859 × 10^9^/L). The serum creatinine level among the cases (1.11 ± 1.64 mg/dl) was comparable to the controls (1.66 ± 1.75 mg/dl).

The sensitivities and specificities as well as the rest of the predictive parameters are summarized in Table 2. All assays demonstrated modest sensitivity (80–90%) and specificity (75–90%). The ELISA detecting anti-S protein had slightly superior sensitivity for IgG (88.9%; 95% confidence interval, CI: 82.81–93.39%) and IgM (86.3; 95% CI: 79.79–91.30%) in comparison to the CLIA assays detecting the total antibodies (82.4%) or IgG (76.5–85.6%).

**Table 2:** Diagnostic statistics of the serological assays

Diagnostic tests		Sensitivity	Specificity	PPV	NPV	Positive likelihood ratio	Negative likelihood ratio	Accuracy
ELISA	Inbios IgG	88.9%	82.0%	83.4%	87.9%	5.13	0.13	85.81%
	Inbios IgM	86.3%	76.7%	79%	84.6%	5.39	0.16	85.15%
CLIA	Siemens T	82.4%	74.7%	76.8%	80.6%	3.09	0.24	77.89%
	Siemens G	76.5%	86.7%	85.4%	78.3%	5.64	0.29	80.86%
	Roche	85.6%	82.0%	82.9%	84.8%	4.76	0.18	83.83%
	Abbott	83.0%	89.3%	88.8%	83.8%	6.92	0.19	85.48%
	DiaSorin	83.7%	82.0%	82.6%	83.1%	4.18	0.20	81.85%

Overall, the sensitivities of CLIA assays were very similar, though Roche IgG showed the best results (85.6%) followed by Abbott and Liaison, both of which had sensitivities of 83%. Abbot showed the best specificity of 89.3%; whereas the specificities of Liaison and Roche were 82%. The best PPV was observed for Abbott IgG CLIA (88.8%). The sensitivity of Siemens IgG was lower (76.5%; 95% CI: 67.54–81.79%) than that of Siemens total (82.4%; 95% CI: 75.37–88.04%).

Regarding the accuracies, Inbios IgG and IgM ELISA were comparatively better than any of the CLIA evaluated in this study. The Inbios IgM ELISA had 35 false positives among controls, 17 of which were scrub typhus cases. Of the scrub typhus cases that were Inbios IgM ELISA positive, 10 were pandemic scrub typhus cases with negative COVID RT-PCR and 7 were pre-pandemic scrub typhus cases in whom RT-PCR was not performed. Similarly, Siemens Total antibody tested 38 false positives among controls, 18 of whom had acute febrile illnesses: dengue (5), malaria (3), scrub typhus (9) and typhoid (1).

**Fig. 2:**
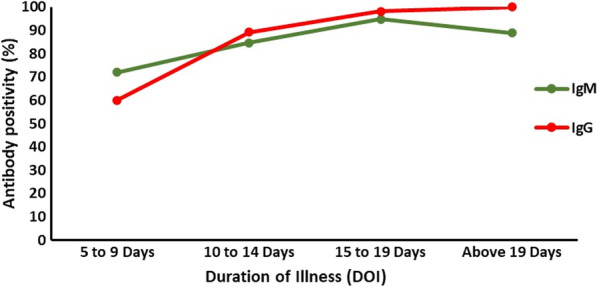
Dynamics of the anti-SARS-CoV-2 S antibody response in COVID-19 cases

The duration of illness had a significant correlation with the antibody response as depicted in Fig. 2. Seven additional patients were diagnosed with COVID-19 using Inbios IgG as compared to IgM ELISA. Among these seven patients, six (66.67%) had a duration of illness of 10 days or more. It was also noted that 8 RT-PCR confirmed COVID-19 cases tested negative to IgM and IgG by all the serological tests. Five of them had duration of illness of 10 days or less, two had duration of illness of 11–14 days, and the duration of illness of one was 17 days. Among all the cases it was noted that the rate of positivity increased with the duration of illness reaching 90% by day 10 to 14. Overall, 129 (84.3%) cases were positive for IgM and 140 cases (91.5%) were positive for IgG either by one or all the assays.

In order to evaluate the performance of the various serological tests for assessment of seroprevalence, the cases were compared against pre-pandemic controls, the result of which is tabulated in Table 3. The specificities of all the serological assays were higher when cases were compared against pre-pandemic controls than when cases were compared against all controls. Siemens IgG, Roche IgG, and Abbott IgG had perfect specificities followed by Inbios IgG ELISA (97.6%). For seroprevalence studies, the Inbios ELISA test detecting anti-S IgG gave the best accuracy 90.8%.

**Table 3:** Diagnostic statistics of the serological assays with Pre-pandemic controls

Diagnostic tests		Sensitivity	Specificity	PPV	NPV	Positive likelihood ratio	Negative likelihood ratio	Accuracy
ELISA	Inbios IgG	88.9%	97.6%	95.6%	63.2%	37.33	0.11	90.77%
	Inbios IgM	86.3%	85.7%	99.3%	70.6%	6.03	0.16	86.08%
CLIA	Siemens T	82.4%	88.1%	96.2%	57.8%	6.92	0.2	83.59%
	Siemens G	76.5%	100%	100%	53.8%	–	0.24	81.54%
	Roche	85.6%	100%	100%	65.6%	–	0.14	88.72%
	Abbott	83.0%	100%	100%	64.6%	–	0.15	88.02%
	DiaSorin	83.7%	95.2%	98.5%	61.5%	17.57	0.17	86.15%

## Discussion

We evaluated seven commercially available serological tests detecting SARS-CoV-2 antibodies—ELISAs detecting the anti-S protein IgM and IgG and CLIA detecting anti N, anti-S IgG, and total antibodies for their diagnostic value. The serological tests detecting anti-S protein IgG and IgM showed modest sensitivity and specificity (80–90%) for diagnosing acute SARS-CoV-2 infection. The diagnostic accuracy of antibody detection by ELISA (85%) was comparable to that of CLIA (80–84%). The tests detecting IgG had near-perfect specificity when cases were compared with pre-pandemic controls. Additionally, ELISA detecting anti-S IgG had the best accuracy for detection of COVID-19 beyond 3 weeks after the onset of symptoms and could be useful for the purpose of seroprevalence studies.

The dynamic pattern of antibodies based on the duration of illness in our study offers important insights. Among patients with duration of illness for 5–9 days, IgM was positive in about 60%, gradually increasing to 90% after day 14 of illness. The sensitivity of IgG assays was lower ranging from 70.8 to 80% within the first 2 weeks from the onset of symptoms. Improving sensitivity with duration of illness has been reported [10–12]. In a meta-analysis evaluating 40 studies, sensitivities ranging from 13.4% to 50.3% were reported within the first week which is much lesser when compared to sensitivity of 69.9–98.9% beyond 3 weeks [13]. A good correlation of serological assays with virus neutralization tests have also been reported [11].

Pending reliable data, serological diagnostics are not currently recommended by the WHO, Pan American Health Organization, or Infectious Diseases Society of America. Our study adds to the emerging data, attempting to fill this important gap. The sensitivity and specificity of *SARS CoV2 Detect IgG* ELISA were found to be 88.9% and 82.7%, respectively, compared to those of the CLIA Roche at 85.6% and 82%, respectively. A recent systematic review which included 39 studies with 11,516 patients reported a similar finding to that of ours with pooled sensitivity of IgG & IgM based ELISA to be 82.9% and 83.8% and CLIA platforms to be 93.1% and 85.1%, respectively [14]. Similar lower sensitivities with CLIA platforms have been reported, especially with duration of illness of less than 14 days [10]. The specificity of CLIA Roche in our study was much lower than that reported by Tan et al. who reported a perfect specificity [15]. While the negative controls used in that study included confirmed viral infections, other closely mimicking acute febrile illnesses were not considered. The same study also evaluated Abbott *SARS CoV2 IgG* CLIA and reported a sensitivity of 84.4% and perfect specificity in comparison to the 83% sensitivity and 89.3% specificity reported in our study. Siemens *SARS CoV2 IgG (COV2G)* and Siemens *SARS CoV2 Total (COV2T)* had sensitivities of 76.5% and 82.4%, respectively, similar to the sensitivities reported recently by Irsara et al. [16].

Less than optimal specificities are an important finding in our study. Seroconversion among the controls during the pandemic period would mostly explain this finding and provides real world accuracy of serology. Additionally, asymptomatic COVID infections, especially among the healthy controls could have biased the diagnostic accuracies of the serological assays. This was probably reflected in the markedly improved specificities when considering cases against only the pre-pandemic controls (Table 3). Siemens IgG, Roche IgG, and Abbott IgG had perfect specificities followed by Inbios IgG ELISA (97.6%). These results are in concordance with the specificities reported by Irsara et al. and as claimed by manufacturers [16].

The positive predictive value among various tests ranged between 76.8% and 88.8%. Hence, the serological assays studied are not ideal for the early diagnosis of COVID-19 within 2 weeks of onset of symptoms. Additionally, this will vary with change in prevalence of the disease in the population tested. The antibody testing may have a role in symptomatic patients with high clinical suspicion of COVID-19 who repeatedly test negative with PCR-based tests, especially after 2 weeks of the onset of symptoms.

The development of antibodies is clearly linked to the severity of the clinical illness [17, 18]. Our study, unlike many from the early pandemic period, included patients with varying severities to avert the bias of reporting diagnostic performance from hospitalized patients with varying severity of disease. Serological tests are less discriminatory in patients with evanescent symptoms, immunocompromised individuals and asymptomatic patients. A heterogeneous antibody response could also be due to the immune status and severity of infection [19].

The duration of illness plays a crucial role in the performance of serological assays [20]. This observation was made in our study as well. Following the initial week of illness, there was a steady increase in the antibodies and the positivity rate of serological assays (Fig. 2). In the initial 7 days of fever, serological tests may remain negative until enough antibodies are formed to make it detectable by assays. However, as the duration of illness progresses, antibodies increase and viral loads decline. Hence, in patients with prolonged symptoms and a negative RT-PCR for SARS-CoV-2 should prompt antibody testing.

Our study highlights the need to continue diagnostic studies in clinical settings since the analytical sensitivity of serological assays may be overestimated by the manufacturers or the Federal Drug Administration for Emergency Use Authorization [21, 22]. A realistic diagnostic accuracy is of particular importance in resource-limited and remote settings and in emergency departments, where rapid diagnosis and prompt initiation of appropriate medication can prove lifesaving [23].

Beyond establishing the diagnosis, accurate serological testing can also help in tracing potential contacts and performing sero-epidemiological studies to assess the burden of infection in the population. While the initial excitement on the concept of “immune passports” was short-lived, serological studies can give an accurate estimate of population immunity and aid in planning vaccine strategies and other control measures. Both Roche CLIA and Inbios IgG ELISA with their superior accuracy can be employed in seroprevalence studies. These results have been replicated in studies utilizing other commercially available immunoassays as well [24]. CLIAs are reported to be capable of handling large sample volumes; but they are expensive and require expertise. Large-scale studies evaluating the role of lateral flow assays in resource-limited and community-based settings are urgently needed.

This study has few limitations. Asymptomatic COVID-19 infections in a small proportion of healthy controls could be a possibility as the gold standard test was not performed on them which is an important limitation of this study. If present in large numbers, this might have biased the results. However, given the smaller proportion of less than one-third of all chosen controls being healthy asymptomatic controls, this possibility is minimal. Additionally, our findings are similar to other observations with added insights into the clinical utility of various serological tests. Cross-reactivity with other coronaviruses, a possible limitation, was not evaluated in this study.

## Conclusions

This study provides a large-scale evaluation of various serological tests for diagnosing COVID-19. The study reports comparable diagnostic accuracy with both the ELISA and CLIA platforms. The results highlight the adjunctive role of serological tests for diagnosis of COVID-19 in patients with persistent symptoms, especially in the second week of illness and beyond. In addition, they are useful in seroprevalence studies which would generate valuable data crucial to contain the pandemic.

## Data Availability

De-identified patient data can be shared to researchers upon request to bmplii@cmcvellore.ac.in after providing suitable justification which will be subject to approval from the COVID-19 Core Research Committee and Ethics Committee of Christian medical college, Vellore.

## Abbreviations

CI: Confidence interval
CLIA: Chemiluminescence immunoassay
COVID-19: Coronavirus Disease 2019
ELISA: Enzyme-linked immunosorbent assay
IgA: Immunoglobulin A
IgG: Immunoglobulin G
IgM: Immunoglobulin M
LFA: Lateral Flow immunoassays
NP: Nucleocapsid protein
NPV: Negative predictive value
PPV: Positive predictive value
RBD: Receptor binding domain
RNA: Ribosomal nucleic acid
RTPCR: Reverse transcription polymerase chain reaction
SARS-CoV-2: Severe acute respiratory syndrome coronavirus-2
TC: Total count
WHO: World Health Organisation
